# BeEP Server: using evolutionary information for quality assessment of protein structure models

**DOI:** 10.1093/nar/gkt453

**Published:** 2013-05-31

**Authors:** Nicolas Palopoli, Esteban Lanzarotti, Gustavo Parisi

**Affiliations:** ^1^Departamento de Ciencia y Tecnologia, Universidad Nacional de Quilmes, B1876BXD, Bernal, Buenos Aires, Argentina, ^2^Centre for Biological Sciences, University of Southampton, SO17 1BJ, Southampton, UK and ^3^Departamento de Quimica Biologica, Facultad de Ciencias Exactas y Naturales, Universidad de Buenos Aires, C1428EHA, Buenos Aires, Argentina

## Abstract

The BeEP Server (http://www.embnet.qb.fcen.uba.ar/embnet/beep.php) is an online resource aimed to help in the endgame of protein structure prediction. It is able to rank submitted structural models of a protein through an explicit use of evolutionary information, a criterion differing from structural or energetic considerations commonly used in other assessment programs. The idea behind BeEP (Best Evolutionary Pattern) is to benefit from the substitution pattern derived from structural constraints present in a set of homologous proteins adopting a given protein conformation. The BeEP method uses a model of protein evolution that takes into account the structure of a protein to build site-specific substitution matrices. The suitability of these substitution matrices is assessed through maximum likelihood calculations from which position-specific and global scores can be derived. These scores estimate how well the structural constraints derived from each structural model are represented in a sequence alignment of homologous proteins. Our assessment on a subset of proteins from the Critical Assessment of techniques for protein Structure Prediction (CASP) experiment has shown that BeEP is capable of discriminating the models and selecting one or more native-like structures. Moreover, BeEP is not explicitly parameterized to find structural similarities between models and given targets, potentially helping to explore the conformational ensemble of the native state.

## INTRODUCTION

In recent years, the protein structure prediction community has dedicated great efforts to predict more accurate structural models of proteins lacking known NMR- or crystallography-solved structures. Their achievements and steady progress have been recorded mainly in the biennial Critical Assessment of techniques for protein Structure Prediction (CASP) experiments (1–3). Although CASP contains several parallel experiments, an important part of it consists of asking the scientific community for structural predictions of selected protein targets, which at the moment of the experiment have already been solved but not yet released to the public. After the deadline for model submission is reached, the models uploaded by each participating group are compared with the newly released experimental structures for the corresponding target protein. Using different measures of structural similarity (4), the models are ranked according to how well they resemble the corresponding target structure, concurrently evaluating the different methods that were applied to generate the models.

The main reason these efforts are being carried out is the close relationship between protein structure and biological function, which has enormous impact in fields such as genomics, proteomics and biotechnology. However, it is broadly overlooked that protein function is more related to protein dynamism than a single protein structure(5,6). Under this view, the native state of proteins is not unique and is better described by an ensemble of conformers in equilibrium, a key concept in the understanding of protein function (7), catalytic processes in enzymes (8), protein–protein recognition (9) and the origins of new biological functions (10).

Besides the important progress in protein modelling, model quality assessment and model selection reported in the last few years(11), the next steps needed to improve quality assessment could be related to the development of tools taking into account the conformational ensemble describing the native state. It has been noted that the extension of structural dissimilarities between conformers could be important in the universe of protein folds (12,13). Although a thorough structural comparison of conformers with solved structures shows a distribution of pairwise RMSD values with a peak around 0.3 Å, this distribution has a large skew to higher values of RMSD reaching maximums above 20 Å of RMSD (12). The fact that the native state of a protein could be represented by such different conformers could suggest that quality assessment protocols that rely heavily on structural comparisons, like those applied in the CASP experiment (4) and in the derivation of different model quality assessment programs (14,15), may be biased by unique structures selected as the targets representing native state of proteins.

In this work, we present the web server implementation of a novel method aimed to assess the quality of protein structural models. BeEP, named after *Best Evolutionary Pattern*, relies on the well-established observation that the conservation of protein structure during evolution constrains sequence divergence (16,17). It has been shown that the specific structural arrangements of the different conformers in the native ensemble of a protein contribute unequally through their specific structural arrangements to the global substitution pattern of the evolving protein (18). Using a model of protein evolution that takes into account protein tertiary structure to derive site-specific substitution matrices, BeEP can assess how well this structure model describes the structurally constrained substitution pattern found in a set of its homologous proteins. We found that the BeEP score is able to discriminate good structural models among a set of decoys extracted from the CASP experiment. Because the BeEP score does not rely on any measure of structural similarity against a given target structure, it could potentially select models belonging to the conformational ensemble of the native state even though they show remarkable structural differences.

The BeEP Server is an online implementation of the BeEP method focused on the evaluation of a narrow set of protein structure models in the latest steps of typical prediction approaches. Unlike the standalone version of BeEP, the web server provides a clean interface to the method and an extended graphical output for easier interpretation of the results. The BeEP Server asks the user to provide at least one protein structure file in the typical PDB format. Additional input files (see below) are required and should be uploaded by the user for optimum control of the process but otherwise they would be generated automatically by the server. The outcome is a ranked list of the submitted models according to their BeEP score. The selected model(s) can be downloaded for further exploration along with all relevant data from the analysis. The site-specific scores are presented graphically and mapped on the models for an easy identification of those sites subjected to specific structural constraints, suggesting possible functional hot spots.

## MATERIALS AND METHODS

### Overview of the BeEP method

**Figure 1.**
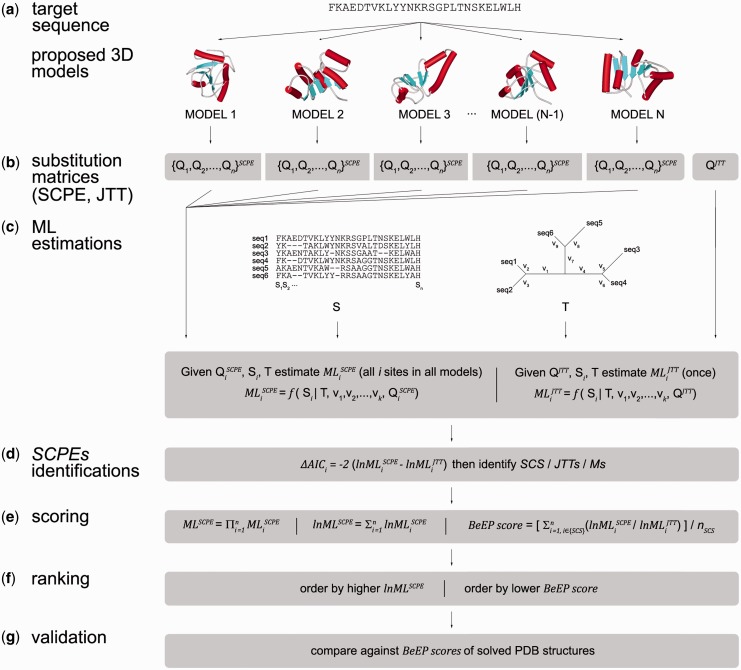
Schematic representation of the BeEP workflow. Given a protein of interest with length n and different proposed structural models (**a**), the SCPE is used to derive a set of site-specific substitution matrices for each model (**b**). Using ML estimations, it is possible to evaluate the correlation of each substitution matrix with the information contained in a sequence alignment S of homologous proteins by optimizing the branch lengths on a corresponding phylogenetic tree T (**c**). The site-specific ML values obtained using SCPE matrices are compared with the ML values calculated with the substitution matrix Q^JTT^ of the unconstrained model JTT to identify sites subjected to structural constraints (SCS) (**d**). BeEP scores are derived from the site-specific ML (**e**) values and the set of structural models can be ranked through the comparison of these scores (**f**). Further validation of native-like models can be achieved by comparison with the BeEP scores of known structures (**g**).

The central idea of the BeEP method is to use the substitution pattern derived from structural constraints during evolution and implicitly contained in a set of homologous proteins (Figure 1). The structural information that can be extracted from a sequence alignment is extensively used in bioinformatics applications to detect close and remote homologous proteins and has been incorporated in sequence-based methods for functional annotation (20–22). More recently, different evolutionary models have been developed to study how the structural constraints modulate protein evolution (23–26). Using a structure-based evolutionary model, it is possible to derive a site-specific substitution pattern for a given structural model and compare this information with the substitution pattern found in an alignment of homologous proteins that adopt the same specific fold. The models can then be ranked depending on how well a given conformation (represented by the site-specific matrices) describes the substitution pattern found in the homologous sequence alignment.

Starting with a target sequence and a set of proposed structural models (Figure 1a), the BeEP method uses the Structurally Constrained model of Protein Evolution (SCPE) (24) to obtain a set of site-specific substitution matrices for a given structural model (27) (Figure 1b). The comparison of this simulated substitution pattern derived from SCPE with the information contained in a set of homologous sequences is performed through a Maximum Likelihood (ML) approach (28) (Figure 1c). We have previously defined the structurally constrained sites (SCS) (18) as those where SCPE significantly outperforms unconstrained evolutionary models that do not contain structural information. The reference model in the BeEP Server calculations is the JTT model (29) (Figure 1d). In a previous analysis of 900 randomly chosen structures, we found an average of 48% of the positions being SCS when conformational diversity is considered (18), suggesting the importance of structural constraints in protein evolution. To statistically test the difference between both models, BeEP calculates the site-specific ΔAIC parameter (30,31). Using the ML for SCPE and JTT models and the number of structurally constrained sites, we defined the BeEP score as:

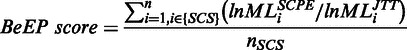



Here, 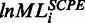 and 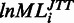 indicate the 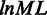 estimation for the position 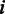 considering the SCPE and JTT models, respectively, 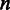 is the length of the protein and 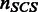 is the number of structurally constrained sites (Figure 1e). Note that the sum is performed over the SCS only. As we previously showed, SCS are generally well conserved during evolution probably because they are associated with the stabilization of the protein fold (18,32,33). The ratio 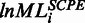
*/*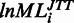 in the BeEP score definition tries to capture how well the specific structural constraints derived from SCPE are represented in the alignment. The normalization by the number of SCS in the BeEP score is necessary because the number of SCS increase with protein size. BeEP score is then used to rank the different models (Figure 1f).

An empirical distribution of BeEP scores was obtained by running BeEP for a dataset of 3192 domain structures randomly taken from different CATH families (34), with lengths between 50 and 450 residues. This distribution gives reference values for native-like BeEP scores that may be useful for an independent estimation of the quality of a single structure model (Figure 1g).

### Performance evaluation

**Figure 2.**
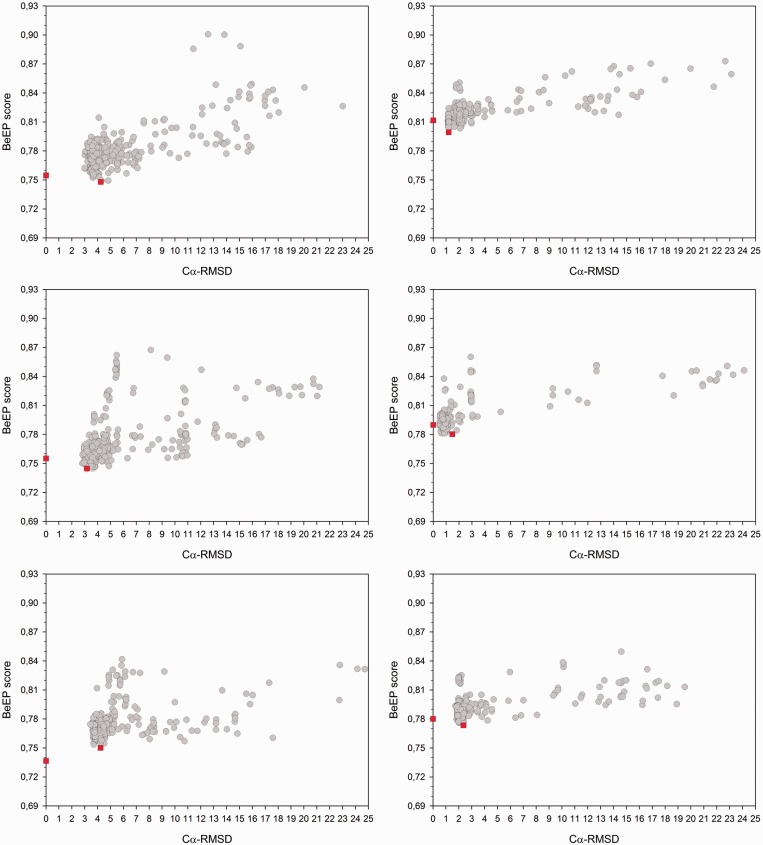
BeEP score versus Cα-RMSD to target protein for all structural models in six example sets selected from CASP8 targets (from left to right and top to bottom: T0411, T0418_D1, T0420, T0426, T0427_D2, T0506_D1). Each grey circle corresponds to a decoy model. The target structure (at Cα-RMSD = 0) and the best decoy are shown in red squares.

**Figure 3.**
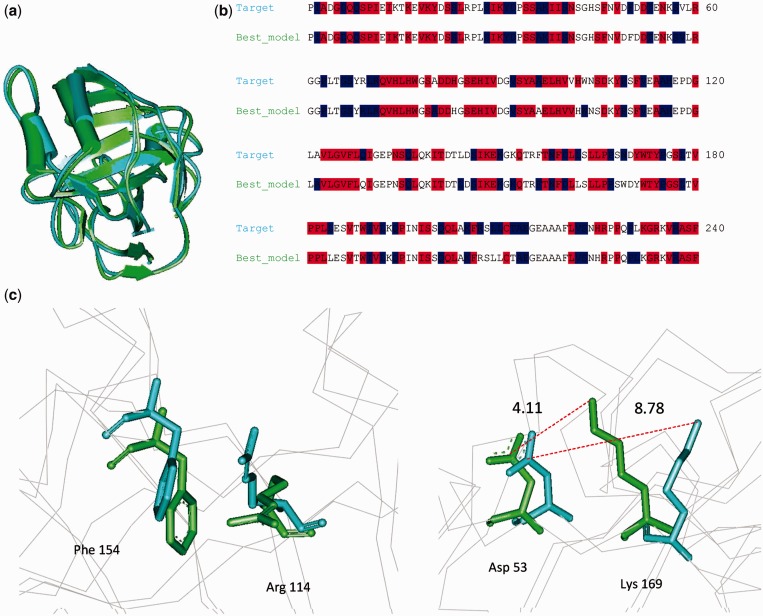
Slight variations in residue environments can change the BeEP score and increase the discrimination of decoys. In panel (**a**), we show the structural alignment between target structure T0426 (cyan) and the best decoy (light green) according to BeEP, which is ranked better than the target itself (see also Figure 2). Structural variations between target and best decoy produce changes in physicochemical environments of the residues favouring SCPE or JTT models. Derived SCPE (in red) and JTT (blue) sites are displayed in panel (**b**). The number of SCS in the target and in the best decoy is 106 and 103, respectively. However, the BeEP score accounts for the difference in the likelihood between SCPE and JTT models in SCS sites (see BeEP score equation in Methods). In panel (**c**), we show two examples of how different residues rearrangements could favour the occurrence of given residues and then increase SCPE likelihood in the best decoy against the target structure. In panel c, left, a pair of arginine (Arg) and phenylalanine (Phe) show a better geometry to form a pi-cation interaction in the best decoy. In panel c, right, the distance to establish a Coulomb interaction between aspartate (Asp) and lysine (Lys) residues is better in the best decoy than in the target structure.

We have tested the BeEP method on a subset of the protein structure models made available for the TS category (Tertiary Structure prediction) in the 8th community-wide experiment on the Critical Assessment of techniques for protein Structure Prediction (CASP8). In general, the CASP experiment is expected to involve heterogeneous sets of good quality structure models, as they are built *ad hoc* following diverse protocols. The predicted decoys were downloaded from the Prediction Center website (http://predictioncenter.org/casp8/results.cgi) while the corresponding edited target PDB structures were recovered from Nick Grishin’s laboratory (http://prodata.swmed.edu/CASP8/evaluation/CASP8Home.htm). Although the BeEP Server can automatically generate the necessary multiple sequence alignments, for this assessment, we derived the alignments from the pre-computed structure–sequence alignments of the HSSP database (35), as the PDB targets are known. These alignments and the phylogenetic trees generated from them are provided as Supplementary File 1. Our final dataset has 55 targets from the Comparative Modelling categories, most of them being single domain proteins of 137 residues on average, with 344 decoys in average per target for a total of 18 955 decoys. The BeEP scores and Cα-RMSD to the reference structure for all decoys in the 55 targets are shown in Supplementary Table S1. Our analysis showed that in most cases, there is a majority of decoys with best (low) BeEP scores that cluster with a low Cα-RMSD to the reference structure. Figure 2 shows some examples of the BeEP score as a function of the Cα-RMSD (similar behavior is observed using other structural similarity measurements), with the scores of the target structure and the best decoy presented in red squares. For all 55 targets used in this study, we found that by selecting the best available decoy, there is a 77% chance overall of picking a decoy that is structurally similar to the target structure, with an average Cα-RMSD of 4.63 Å. We noted that in most cases, the target structure is in the middle of a cloud of low-RMSD decoys where the best decoy is not the one with the minimum Cα-RMSD. Interestingly, slight variations in physicochemical environments could favour that several models perform better than the target itself (Figure 3).

### Input files needed for the BeEP Server

The input for the BeEP Server is at least one protein structure file in PDB format. Additional structure models could be uploaded individually or in compressed files. Two other files are required for BeEP for the ML calculation: a multiple sequence alignment (MSA) of the protein and its homologues and the topology of a phylogenetic tree constructed from the MSA. Although the server can generate these files in an entirely automatic way, curated versions of the MSA and the topology tree should be preferred. The MSA file should be in the Phylip format (35), which can be directly generated from most alignment programs. If the MSA is not provided, it will be built by the server in the following way. First, a set of related sequences is retrieved through five iterations of PSI-BLAST searches (22) with the reference protein against the UniREF90 database (36) using an e-value cut-off of 0.001. The MSA is built from the PSI-BLAST query-hit pairwise results by random selection of no more than 10 sequences for each 10% bin in the range of 30–100% sequence identity, resulting in a MSA of up to 70 sequences. However, in case the alignment has <30 sequences, the selection is performed again but allowing up to 30 sequences for each 10% bin. This filtering of the MSA is necessary to restrict its size and avoid excessively large ML calculations.

The BeEP Server also requires a text file containing a phylogenetic tree constructed from the corresponding MSA. Only the tree topology (the branching pattern) is considered, thus distances between leaves and branch support values may be missing in the tree. The tree might be built by any method of choice, but it must be expressed in the Newick format. Failure to upload this file will make the BeEP Server to generate it from the available MSA using the PROTPARS protein parsimony algorithm version 3.68 from the Phylip package, with default parameters.

### Input check

The BeEP Server extracts the heavy atoms in the backbone and sidechains of the first chain in each PDB file. The server does not allow sets of PDB files with different sequences. It also checks that the PDB sequence is the same as the first sequence of the MSA, that it has no indels and that the number of proteins in the MSA is consistent with the number of leaves in the phylogenetic tree topology. Each user-submitted job is assigned a unique JobID and placed in a queue to be run as soon as possible. The JobID can be used to see the progress of the analysis and retrieve the results when ready.

## RESULTS

### Output of the BeEP Server

**Figure 4.**
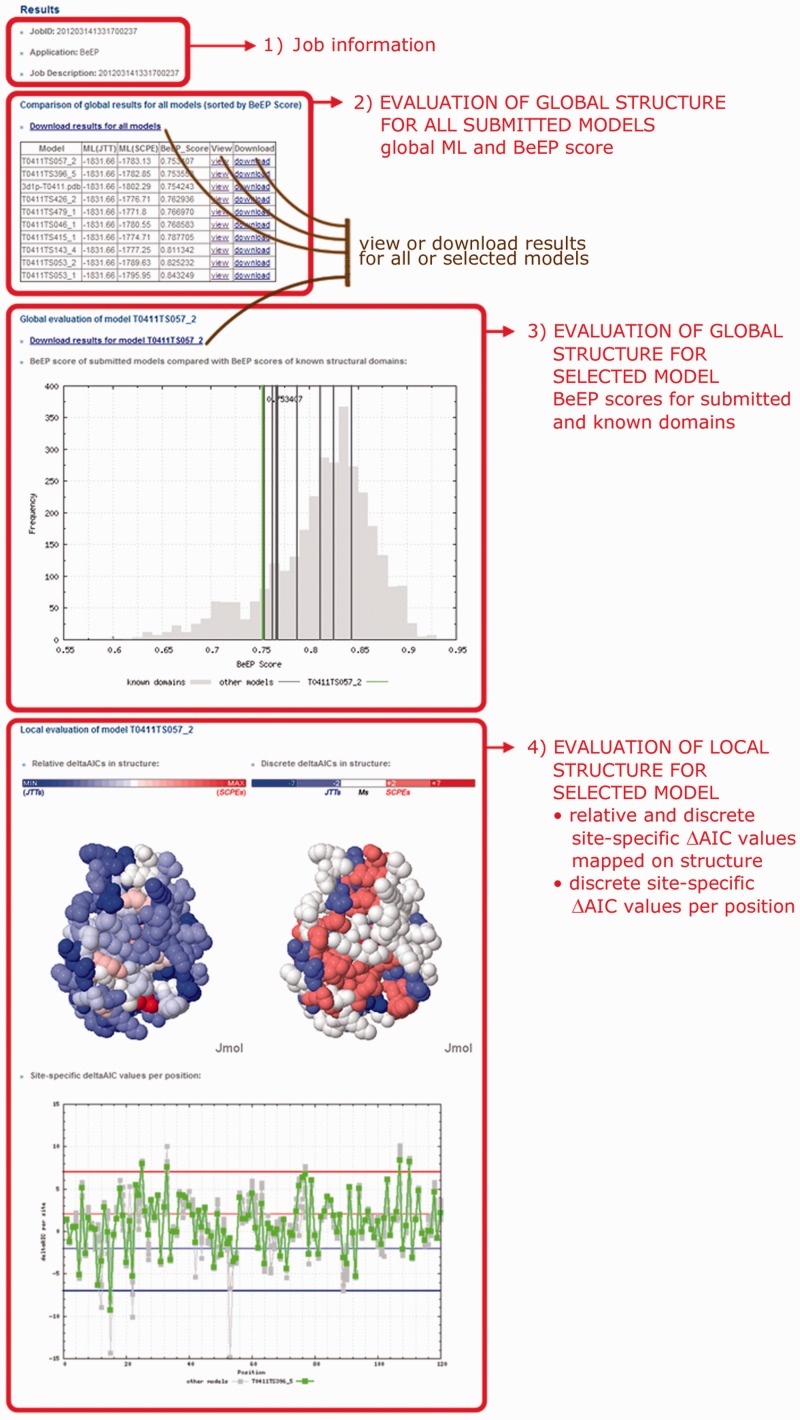
BeEP Server output explanation. The red boxes indicate different sections in the output. 1) Job information. The JobID can be used to retrieve results after the run has finished. 2) Table summarizing results of the global assessment of submitted protein structure models. For each submitted model, the table shows the global ML values obtained with the JTT general substitution model and the SCPE site-specific substitution model, together with the BeEP score on which the table is ranked. Links are provided to download a compressed file with all the results generated by the run, both for the individual models and for the complete dataset, or to load the results for a selected model. 3) BeEP score of all submitted models plotted on top of the distribution of BeEP scores for PDB structures of known domains. The selected model is displayed in green. The BeEP scores of known domains are provided as a reference, with good structure models expected to tend to the left (low) end of the distribution. 4) Different representations of the selected model based on the local ΔAIC values. Site-specific scores are mapped on the structure in two different scales: the discrete colouring is useful for spotting SCS while the relative colouring can point to structurally conserved patches. A plot of site-specific ΔAIC values per position helps to identify contiguous regions of the protein subjected to structural constraints. The reference horizontal lines are coloured according to the scale of discrete ΔAIC values.

The output of the BeEP Server is a number of predictions for each uploaded structure, presented as a table of scores and a series of graphs (see Figure 4). A table listing global scores for all uploaded structures sorted by the BeEP score enables a direct comparison between the structure models. The BeEP scores are displayed on top of the histogram showing the empirical distribution of BeEP scores for a sample of 3191 known PDB domain structures. Following the BeEP score definition, it is expected that good models should tend to the left (low) end of this distribution. The server also provides two different plots based on site-specific ΔAIC values to help interpret the contribution of individual sites to the global score of a selected model. The ΔAIC values are depicted on a sphere representation of the structure using the Jmol browser plugin, with colour schemes that follow either a discrete or a relative scale. On the discrete scale, it is possible to identify the structurally constrained sites, as well as sites where the JTT matrix is preferred (named JTT sites or JTTs) and those where there is no statistical difference between both models (named mutational sites or Ms). The relative scale refers to the compared ΔAIC values of the given structure and is useful for a quick exploration of regions under stronger structural constraints. A plot of the ΔAIC values per position helps to identify stretches of protein sequence under different structural constraints.

The results page allows the user to download compressed files with all input and output files related to the BeEP Server analysis, both for each individual structure and for the combination of them all.

## DISCUSSION

Several quality assessment methods have been developed using different forms of evolutionary information encoded in sequence alignments (37–39). As far as we know, BeEP is one of the first quality assessment methods that incorporates an evolutionary model to explain the substitution pattern derived from structural constraints during evolution.

Our results suggest that the BeEP score could highlight decoys belonging to the conformational ensemble of the native state, which in turn are not necessarily the most similar to the target structure. In our assessment on a set of CASP targets, it is a general trend that the target structure does not display the lowest BeEP score and in general is surrounded by a cloud of low-RMSD decoys.

It is interesting to note that, when exploring substitution patterns, the use of an evolutionary model can be more powerful than the application of a residue conservation approach. Evolutionary models try to reproduce amino acids changes as a function of evolutionary time, while a conservative approach tries to understand the outcome of this process (actual amino acid composition in a given alignment). However, the increase in the reliability of the description of the substitution process is associated with an increase in the computational cost in particular for ML computations. This is a major drawback for online servers, and it is the reason why the BeEP Server is only suitable for the analysis of some tens of models in the final steps of protein structure modelling.

## SUPPLEMENTARY DATA

Supplementary Data are available at NAR Online: Supplementary Table 1 and Supplementary File 1.

## FUNDING

N.P. is a former PhD fellow from CONICET (Consejo Nacional de Investigaciones Científicas y Técnicas) and current Research Fellow at the University of Southampton. E.L. is a PhD fellow from the University of Buenos Aires. G.P. is a researcher from CONICET. Funding for open access charge: National Institutions in Argentina: PIP CONICET [112-200801-02849] and UNQ [1004/11].

*Conflict of interest statement.* None declared.
